# Minimum Information for Reporting on the Comet Assay (MIRCA): recommendations for describing comet assay procedures and results

**DOI:** 10.1038/s41596-020-0398-1

**Published:** 2020-10-26

**Authors:** Peter Møller, Amaya Azqueta, Elisa Boutet-Robinet, Gudrun Koppen, Stefano Bonassi, Mirta Milić, Goran Gajski, Solange Costa, João Paulo Teixeira, Cristiana Costa Pereira, Maria Dusinska, Roger Godschalk, Gunnar Brunborg, Kristine B. Gutzkow, Lisa Giovannelli, Marcus S. Cooke, Elke Richling, Blanca Laffon, Vanessa Valdiglesias, Nursen Basaran, Cristian Del Bo’, Bojana Zegura, Matjaz Novak, Helga Stopper, Pavel Vodicka, Sona Vodenkova, Vanessa Moraes de Andrade, Monika Sramkova, Alena Gabelova, Andrew Collins, Sabine A. S. Langie

**Affiliations:** 1 Department of Public Health, Section of Environmental Health, University of Copenhagen, Copenhagen, Denmark; 2 Department of Pharmacology and Toxicology, University of Navarra, Pamplona, Spain; 3 IdiSNA, Navarra Institute for Health Research, Pamplona, Spain; 4 Toxalim (Research Centre in Food Toxicology), Université de Toulouse, INRAE, ENVT, INP-Purpan, UPS, Toulouse, France; 5 VITO-HEALTH, Mol, Belgium; 6 Unit of Clinical and Molecular Epidemiology IRCCS San Raffaele Pisana, Rome, Italy; 7 Department of Human Sciences and Quality of Life Promotion, San Raffaele University, Rome, Italy; 8 Mutagenesis Unit, Institute for Medical Research and Occupational Health, Zagreb, Croatia; 9 Environmental Health Department, National Institute of Health Dr Ricardo Jorge, Porto, Portugal; 10 Epidemiological Research Unit (EPIUnit), Instituto de Saúde Pública, Universidade do Porto, Porto, Portugal; 11 Health Effects Laboratory, Department of Environmental Chemistry, Norwegian Institute for Air Research (NILU), Kjeller, Norway; 12 Department of Pharmacology & Toxicology, School for Nutrition and Translational Research in Metabolism (NUTRIM), Maastricht University, Maastricht, the Netherlands; 13 Section of Molecular Toxicology, Department of Environmental Health, Norwegian Institute of Public Health Skøyen, Oslo, Norway; 14 Department of Neuroscience, Psychology, Pharmacology and Child Health (NEUROFARBA), Section Pharmacology and Toxicology, University of Florence, Florence, Italy; 15 Oxidative Stress Group, Department of Cell Biology, Microbiology and Molecular Biology, University of South Florida, Tampa, FL USA; 16 Food Chemistry & Toxicology, Department of Chemistry, University of Kaiserslautern, Kaiserslautern, Germany; 17 Universidade da Coruña, Grupo DICOMOSA, Centro de Investigaciones Científicas Avanzadas (CICA), Departamento de Psicología, Facultad de Ciencias de la Educación, Coruña, Spain; 18 Instituto de Investigación Biomédica de A Coruña (INIBIC), AE CICA-INIBIC, Coruña, Spain; 19 Department of Toxicology, Faculty of Pharmacy, Hacettepe University, Ankara, Turkey; 20 Division of Human Nutrition, Department of Food, Environmental and Nutritional Sciences (DeFENS), Università degli Studi di Milano, Milan, Italy; 21 Department of Genetic Toxicology and Cancer Biology, National Institute of Biology, Ljubljana, Slovenia; 22 Institute of Pharmacology and Toxicology, University of Würzburg, Würzburg, Germany; 23 Institute of Experimental Medicine, Czech Academy of Sciences, Prague, Czech Republic; 24 Biomedical Center, Medical Faculty in Pilsen, Charles University in Prague, Prague, Czech Republic; 25 Laboratory of Translational Biomedicine, University of Southern Santa Catarina, UNESC, Criciúma, Brazil; 26 Department of Nanobiology, Cancer Research Institute, Biomedical Research Center of the Slovak Academy of Sciences, Bratislava, Slovakia; 27 Department of Nutrition, University of Oslo, Oslo, Norway; 28 Centre for Environmental Sciences, Hasselt University, Hasselt, Belgium

**Keywords:** Biomarkers, Risk factors

## Abstract

The comet assay is a widely used test for the detection of DNA damage and repair activity. However, there are interlaboratory differences in reported levels of baseline and induced damage in the same experimental systems. These differences may be attributed to protocol differences, although it is difficult to identify the relevant conditions because detailed comet assay procedures are not always published. Here, we present a Consensus Statement for the Minimum Information for Reporting Comet Assay (MIRCA) providing recommendations for describing comet assay conditions and results. These recommendations differentiate between ‘desirable’ and ‘essential’ information: ‘essential’ information refers to the precise details that are necessary to assess the quality of the experimental work, whereas ‘desirable’ information relates to technical issues that might be encountered when repeating the experiments. Adherence to MIRCA recommendations should ensure that comet assay results can be easily interpreted and independently verified by other researchers.

## Abstract (web summary)

Here, members of the hCOMET COST Action program provide a consensus statement on the Minimum Information for Reporting Comet Assays (MIRCA).

## Introduction

The alkaline comet assay is a technically simple, sensitive assay to detect DNA damage (strand breaks and other lesions that are converted into strand breaks under alkaline conditions) and DNA repair activity^1–4^. However, international ring trials have identified substantial variations in comet assay procedures and primary descriptors (such as %DNA in tail) between laboratories. This variation could hamper inter-laboratory data comparison and interpretation, as well as attempts to standardize methods and promote the use of reference standards^5–14^. A recent review summarized the plenitude of procedure descriptions and technical recommendations for comet assays that have been published in the past 20 years, and highlighted the problem of inter-laboratory variation in DNA damage levels^15^. The main issue is that the comet assay does not directly measure the number of specific DNA lesions, but rather measures the migration of DNA in agarose gels as a result of the relaxation produced by strand breaks under alkaline conditions. Certain steps in the assay procedure are more important determinants of DNA migration than others. However, published comet assay studies often inadequately describe assay conditions. Even more troubling is the lack of data on positive experimental controls and positive assay controls, which are necessary for assessing assay performance^16,17^.

The OECD guideline on the in vivo comet assay (TG489), which was developed by multiple authors at various different institutions, is the most authoritative set of recommendations for the reporting of in vivo comet assay procedures and results^18^. However, it does not cover in vitro experiments and biomonitoring studies, or endpoints other than DNA strand breaks, and the level of detail is limited in some aspects. For example, the guidelines recommend that “electrophoresis conditions” should be reported but without specifying that these should include the composition and temperature of the electrophoresis solution, as well as the duration of electrophoresis and strength of the field. There is therefore an urgent need for a more comprehensive set of recommendations to describe comet assay conditions, including explanations for why certain steps need to be reported in detail. Similar recommendations have been published for other types of assays, such as the MIAME guidelines for microarrays^19^ and the MIQE guidelines for quantitative real-time polymerase chain reaction procedures^20^. These have been widely adopted by authors and journals, and appear to have improved the harmonization of reporting and the reliability of the results.

The goal of the Minimum Information for Reporting Comet Assay (MIRCA) recommendations is to highlight key aspects of the comet assay procedure that must be described when reporting the results from cell culture studies, animal models, invertebrates, plants and human biomonitoring and clinical studies. We acknowledge that every step in the protocol is important for a well-functioning method. However, the purpose of the MIRCA recommendations is to ensure that specific information about the comet assay procedure is available to readers that will allow the results to be interpreted critically and compared with those from other studies. We have restricted the MIRCA recommendations to the technical performance of the assay. There are other fundamental aspects of conducting and reporting scientific studies (e.g., blinding of samples, minimizing bias and confounding factors, reporting brands and suppliers of chemicals, kits and laboratory equipment, ensuring an appropriate number of replicates and method of statistical analysis, etc.), but these are not unique to comet assays and are not discussed in detail here. For the use of the comet assay in molecular epidemiology studies, we recommend reading the Strengthening the Reporting of Observational Studies in Epidemiology–Molecular Epidemiology (STROBE-ME) statement and following the recommendations there for reporting biomarker results^21^.

### Overview of MIRCA recommendations

The MIRCA recommendations focus on the steps in the comet assay that may affect the level of DNA migration (e.g., a physical effect that causes DNA to move faster in agarose irrespective of the absolute amount of DNA damage) or increase the difference in DNA migration between unexposed and exposed specimens (i.e., detection of induced DNA damage). The variation in comet assay procedures has been discussed previously^15^ and will not be described in detail here. Most comet assays determine DNA damage as frank strand breaks (and alkali-labile sites, which are subsequently converted to strand breaks) by the standard alkaline procedure. However, DNA repair enzymes from bacteria or human cells can also be used to gain further information on specific classes of DNA lesions such as oxidation or alkylation products. The two most commonly used modified comet assay formats for DNA repair are (i) the ‘cellular DNA repair assay’, in which the accumulation and removal of DNA damage is followed over time, and (ii) the ‘comet-based in vitro DNA repair assay’, which measures the DNA incision activity of a cell-free protein extract on substrate DNA (in the form of nucleoids) containing DNA lesions. The ‘neutral comet assay’ (whereby electrophoresis is carried out in a solution of neutral pH) is rarely used^22,23^ and is therefore not included as a separate protocol step in the MIRCA recommendations.

**Fig. 1:**
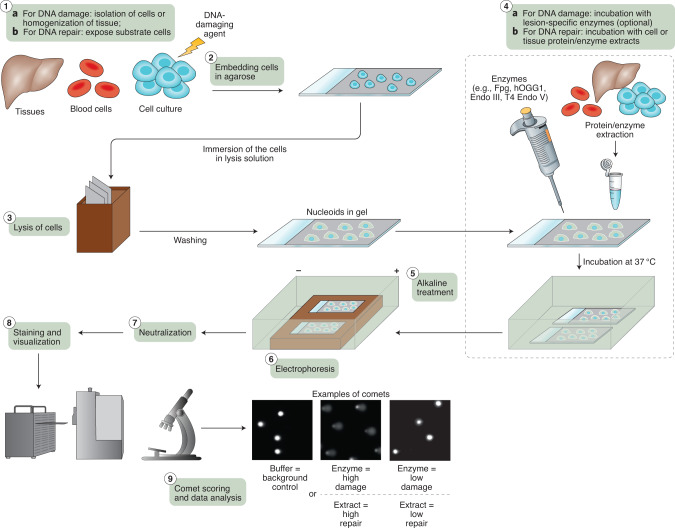
Scheme of the comet assay procedure. The comet assay consists of nine steps, which are described in the text and in Table 1. Tissues or cells are isolated and processed to a single-cell suspension, either to study DNA damage (Step 1A) or to prepare substrate cells for the in vitro DNA repair assay (Step 1B). Next, single cells are embedded in agarose gels (Step 2) and lysed (Step 3) to remove membranes and other cellular material, leaving protein‐depleted nuclei with supercoiled DNA (called ‘nucleoids’). Step 4 does not apply to the standard alkaline comet assay, but comprises specific steps for the enzyme-modified comet assay (i.e., incubation of the nucleoids with lesion-specific enzyme; Step 4A) or in vitro DNA repair assay (i.e., incubation of the substrate cell nucleoids with cell or tissue extract; Step 4B). For the enzyme-modified comet assay, possible enzymes include formamidopyrimidine DNA glycosylase (Fpg), human oxoguanine DNA glycosylase (hOgg1), endonuclease III (Endo III) and T4 endonuclease V (T4 Endo V). In Step 5, samples are treated in alkaline solution to convert alkali-labile sites to strand breaks. The samples are then subjected to alkaline electrophoresis, resulting in the formation of single-cell comets (Step 6), and then rinsed in neutralizing solution (Step 7). Step 8 includes staining and visualization of the comets by fluorescence microscopy. Examples of comets include nucleoids that have been incubated with buffer in Step 4 (i.e., DNA strand breaks or background control for the enzyme-modified comet assay and in vitro DNA repair assay, respectively). Nucleoids may show clear comet formation if the DNA in the sample contains many lesions or the cell extract has high repair activity. Conversely, sparse DNA lesions and low repair activity give rise to comets that are no different from those for the background control. The comets are then scored, and finally data analysis is performed (Step 9).

Irrespective of the cell or tissue sample being investigated, the comet assay has up to nine steps (Fig. 1), as follows: (i) isolation of cells and preparation of single-cell suspensions, (ii) embedding of the cells in agarose, (iii) cell lysis, (iv) incubation of the nucleoids with lesion-specific enzyme (for the enzyme-modified comet assay) or with cell or tissue extract (for the in vitro DNA repair assay), (v) alkaline treatment, (vi) electrophoresis, (vii) neutralization, (viii) staining and visualization and (ix) scoring and data analysis. Table 1 outlines the MIRCA recommendations for these individual steps. Each recommendation is classified as either ‘desirable’ information or ‘essential’ information to be reported in articles that include comet assay results: ‘essential’ information is necessary for assessing the quality of the comet assay experiments, whereas ‘desirable’ information is only needed for repeating the experiment.

**Table 1:** Reporting checklist for comet assay studies

Step	Comet assay parameter	Reporting requirement^a^	Notes and rationales
1A	**Isolation of cells**		
	Preparation of a single-cell suspension (from solid tissue or cell culture)	Desirable	The homogenization procedure (whether in buffer or medium) may affect levels of DNA damage.
	Cell type	Essential	For human biomonitoring studies, it should be specified whether the samples are whole blood (i.e., with erythrocytes), isolated leukocytes or peripheral blood mononuclear cells, or from which organ or tissue the cells are derived (buccal, sperm, etc.).
	Method for venipuncture and isolation of cells from blood (if cells were isolated)	Desirable	Expected to be of little importance in most cases, but the gauge of needle and anticoagulant used might affect the level of DNA damage during cell isolation.
	Temperature and duration of transfer from isolation of cells to processing of cells	Essential	The temperature and time period between isolation of cells and direct processing in comet assay (or cryopreservation) may affect the level of DNA damage.
	Storage (in case of specimens that have been cryopreserved)	Essential	The freezing and thawing procedures might increase the basal level of DNA migration. For clinical intervention studies, it is essential to know whether samples taken at different times were analyzed fresh (i.e., in different experiments) or in the same comet assay experiment in the case of cryopreserved samples.
1B	**Substrate cells (for DNA repair assay only)**		
	Substrate cell type	Desirable	The DNA content and chromosome structure differ between different immortalized cells (cell lines) and between primary and immortalized cells.
	Cell density	Desirable	The in vitro DNA repair assay measures the rate of incisions, where the amount of enzyme is the limiting factor. Theoretically, if the DNA migration in each comet depends on number of incisions, increasing the cell density will dilute the effect by yielding fewer incisions per comet.
	Type of exposure used	Essential	Very few (if any) genotoxic agents give rise to DNA lesions that are repaired by only one DNA repair pathway; rather, most give rise to a spectrum of DNA lesions. The concentration/dose of the genotoxic agent should be reported.
	Levels of lesions in the substrate cells	Desirable	It is desirable to know the total number of lesions in the substrate cells because the repair incision activity must be measured under conditions in which the concentration of substrate (lesions) is not rate limiting (in keeping with basic enzymology).
	Storage (in case specimens have been cryopreserved)	Essential	See same item under section 1A.
1C	**Assay controls**		
		Essential	Assay controls should always be included and reported in studies that do not have a positive control group.
1D	**Negative and positive controls**		
		Desirable	Control groups are desirable (or even essential in certain cases). For most purposes, however (and especially in human biomonitoring), assay controls can replace negative and positive controls (i.e., control groups).
2	**Embedding the cells in the agarose**		
	Description of the type of slides	Desirable	Use of 2-gel versus 12-gel format, etc., might affect the level of DNA migration.
	Final concentration of low-melting-point-agarose containing cells	Essential	The final concentration (percentage after the cells have been added) is very important. As the concentration will change upon reuse of the agarose stock solution, it should be specified if it is used more than once. It is not informative enough to state the concentration of the stock solution.
3	**Lysis**		
	Buffer composition	Essential	For buccal cells, an extra lysis step with proteinase K is needed. Lysis of sperm requires an incubation step with dithiothreitol and proteinase K to break disulfide bonds in the tightly packed DNA.
	Duration	Desirable	The duration of the lysis can vary depending on the cell type. If it is too long, this may affect certain types of DNA lesions (e.g., conversion of alkali-stabile lesions to strand breaks), and if too short, lysis might be incomplete. It is important that the same duration is used in all experiments.
	Temperature	Desirable	Expected to have little effect on DNA migration, except in certain cases where alkali-stabile lesions may be converted to strand breaks.
4A	**Enzyme treatment**		
	Washing step between the lysis and the enzyme treatment	Desirable	The enzymes may be inactivated by carryover of the lysis solution, due to high pH and detergents. The composition of the wash buffer should be specified.
	Source of repair enzyme	Essential	There are different manufactures of enzymes for the comet assay, which can be obtained as crude extracts or purified enzymes. Thus, the enzyme activity may vary between manufactures.
	Optimization of enzyme concentration and duration of incubation	Desirable	Authors should report or reference the results from a titration experiment using the same gel incubation unit and mode of incubation as the test samples.
	Duration	Essential	The number of repair incisions is proportional to the incubation time. However, prolonged incubation times may lead to nonspecific incisions.
	Incubation temperature	Essential	The rate of enzymic reactions depends on the temperature.
	Concentration of enzyme applied onto gels	Essential	The amount of enzyme on the gel will affect the number of repair incisions. It is important to report the results from optimization experiments (i.e., the amount of enzyme and the duration of the incubation period).
	Type of incubation unit	Desirable	Incubation in a regular incubator, slide moat or 12-gel system can all give different results.
	Mode of incubation	Desirable	The treatment is done either by dropping the enzyme solution onto the gels and covering with a coverslip or by immersing the slide in the enzyme solution. Differences in resulting enzyme activity have been noted, although this has not been assessed in a systematic manner.
4B	**Extract preparation/incubation (for DNA repair only)**		
	Number of cells or milligrams of tissue used to prepare the extract	Desirable	The number of cells or weight of tissue used to obtain a suitable protein or cell concentration in the enzyme reaction is not directly linked to the repair incisions because further dilutions of the crude protein extract occur, but is a useful indicator of relative amount of activity.
	Protein concentration or cell density in the final extract	Essential	Protein concentration directly affects the rate of repair incisions, so this information should be reported in articles. However, extracts from single-cell suspensions (e.g., blood samples or cell cultures) can be standardized to the same number of cells before the extraction of protein; the cell density in the final repair extract will then be equivalent to the dilution of the same starting cell number.
	Extraction buffer composition	Desirable	This is not likely to affect the repair activity as repair inhibitors are avoided in the buffer.
	Incubation buffer composition	Essential	Essential cofactors and buffer content may affect the activity of the repair enzymes.
	Optimization of enzyme concentration and duration of incubation	Desirable	See same item under section 4A.
	Volume of extract added to the gel	Desirable	Relevant for those who wish to repeat the experiment.
	Duration of incubation	Essential	See same item under section 4A.
	Temperature of incubation	Essential	See same item under section 4A.
	Mode of incubation	Desirable	See same item under section 4A.
	Type of enzyme used as positive assay control	Essential	It is important to clarify the type of enzyme because they have different substrate specificities (e.g., Fpg and hOGG1 do not possess the same lesion specificity).
	Negative assay or background controls	Essential	It is crucial to demonstrate that the repair incisions are not just a result of nonspecific (background) damage to the DNA.
5	**Alkaline treatment**		
	Composition	Essential	The composition of the solution can affect the conversion of alkali-labile sites to DNA strand breaks. The pH value is typically controlled by the amount of NaOH.
	Duration	Essential	Prolongation of the treatment can increase the conversion of alkali-labile sites to DNA strand breaks.
	Temperature	Essential	The temperature will affect the separation of DNA strands.
6	**Alkaline electrophoresis**		
	Composition	Essential	The extent of DNA migration depends on the chemical composition.
	Voltage/cm over the slide support platform	Essential	The extent of DNA migration is directly proportional to the strength of the electrophoretic field.
	Duration	Essential	The extent of DNA migration is directly proportional to the duration of electrophoresis. The duration is restricted to avoided overlap between comets.
	Temperature	Essential	Electrophoresis at high temperature might induce DNA strand breaks and thereby increase the level of DNA migration.
7	**Neutralization**		
	Composition	Desirable	No expected effect on DNA migration.
8	**Staining and visualization**		
	Type of DNA dye	Essential	Dyes have different binding affinity to DNA and may therefore affect the calculation of primary comet assay descriptors in the image analysis software.
	Concentration of dye	Desirable	Most likely does not affect the image analysis of the comets, but desirable information for researchers who want to repeat the specific protocol.
	Time from staining until microscopy	Desirable	Certain dyes may require incubation to produce a good fluorescent signal.
	Microscope magnification	Desirable	For image analysis by software, the DNA migration differs between magnifications.
	Representative images of comets	Desirable	As the calculation of the %DNA in tail (or other descriptor) may be different between different image analysis systems, it is desirable to include images of comets and the level of DNA migration (e.g., as supplementary material or a citation to an earlier article with representative images, or by including images within figures).
9A	**Scoring and data analysis**		
	Type of primary comet assay descriptor	Essential	There are different ways to measure the level of DNA migration (i.e., %DNA in tail, tail length, tail moment and visual score). These primary comet assay descriptors have different scales, which cannot be directly compared.
	Number of comets scored per gel and number of gels scored	Essential	Important because of low precision in the measurement of DNA in gels with few comets.
	Measure of the central value of comet scores (e.g., mean or median when image analysis systems have been used for analysis of DNA migration)	Essential	Using the mean versus median level of DNA migration might affect the estimate of DNA damage, depending on the distribution of comet scores. It is essential that authors clarify that mean/median values from comet distributions come from independent observations (i.e., different animals or humans, or cell culture experiments carried out on different days).
	Type of software for image analysis	Essential	Different software may have different algorithms for calculating primary comet assay descriptors.
	Calibration	Desirable	The primary comet assay descriptor is a relative value (e.g., %DNA in the comet tail). Transformation to lesions per nucleotide or unaltered nucleobase pair is desirable for ease of comparisons between studies, although it does not affect the quality of the comet assay analysis.
9B	**Calculation of enzyme-sensitive sites and DNA repair activity**		
	Calculation of enzyme-sensitive sites	Essential	Results for the enzyme-modified comet assay should be reported as the net increase (i.e., enzyme-treatment with the ‘no enzyme’ level of DNA strand breaks subtracted).
	Calculation of DNA repair activity	Essential	Results for DNA repair activity should be reported as the net incisions (i.e., repair extract treatment with the background level of DNA migration subtracted).
9C	**Statistical analysis of results**		
		Essential	The statistical analysis should conform to standard practice for parametric, nonparametric or logistic regression, depending upon the study design.

^a^Information on each comet assay step is classified as either ‘desirable’ or ‘essential’ information based on a threshold of ≥75% agreement between the authors of this Consensus Statement.

## Methods

The MIRCA guidelines have been crafted by members of hCOMET COST Action with the goal of improving the analysis and reporting of comet assay results (http://www.hcomet.eu/). The authors are comet assay experts with a minimum of eight years of experience with these assays (15 of the authors have >20 years of relevant experience). We have used an internet-based questionnaire to sample opinions from the authors concerning the importance of reporting details (Table 1; Supplementary Data). Each piece of information was graded by the authors as ‘essential’, ‘desirable’ or ‘not important’, and a threshold of ≥75% congruence was used for the classifications. If the number of ‘essential information’ replies did not reach the threshold of 75% agreement, the ‘essential information’ and ‘desirable information’ replies were combined, and the piece of information was classified as ‘desirable information’ if ≥75% authors agreed it was either ‘essential’ or ‘desirable’. In Table 1, we include explanatory notes for each recommendation.

### Specific MIRCA recommendations for each step of the comet assay

#### Step 1A: Isolation of cells and preparation of single-cell suspensions

As the comet assay uses suspensions of single cells, specimens that are not obtained as single cells must be treated mechanically or enzymatically to disrupt the attachment of cells to an extracellular matrix or to each other. The homogenization of tissues by mechanical disruption may itself cause DNA damage, whereas the enzymatic digestion of tissue may lead to the removal of DNA lesions due to the activity of endogenous DNA repair enzymes, or increase DNA damage levels by releasing nucleases from the cells. The composition of the homogenization buffer is ‘desirable’ information, particularly in regard to the components that are necessary for preserving DNA integrity (e.g., ethylenediaminetetraacetic acid)^24,25^. A description of the procedure for the isolation of single-cell suspensions, including homogenization of the tissue, is considered to be ‘desirable’ information to report in articles.

Blood samples are often used in human biomonitoring studies. Because blood represents a heterogeneous population of cells, detailed information on the cell population is ‘essential’ in publications. The text should precisely describe whether the specimens are whole blood (including red blood cells), leukocytes, mononuclear blood cells or a subset of cells (e.g., lymphocytes). It can also be helpful to include information on the procedure for venipuncture, type of anticoagulant and subsequent method of cell isolation (i.e., centrifugation and washing steps) for those repeating the experiment, so this is classified as ‘desirable’ information. Information on the storage conditions of the cells or tissues if they are cryopreserved, as well as the freezing method (e.g., snap freezing on dry ice or in liquid nitrogen, or a slow freezing procedure) and thawing procedure, is considered ‘essential’ information to report, as these processes have been shown to affect the basal level of DNA migration^26^.

#### Step 1B: Preparation of substrate cells for the in vitro DNA repair assay

Any eukaryotic cell type can be used as a substrate cell for the in vitro DNA repair assay, and the cell type and density are ‘desirable’ information to report. It is ‘essential’ to report the method and type of genotoxic compound used to induce DNA lesions in the substrate cells and subsequent cell storage conditions, whereas the total level of DNA lesions that can be detected in the substrate cells (e.g., the number of formamidopyrimidine DNA glycosylase-sensitive sites in cells treated with Ro19-8022 plus light) is considered to be ‘desirable’ information only.

#### Step 1C: Assay controls

In this article, assay controls refer to samples that are included in every comet assay experiment; these are sometimes called reference standards, internal controls or technical controls^16,27^. The assay controls are typically cryopreserved aliquots from a single batch of cells that have been exposed to a DNA strand-breaking agent (e.g., ionizing radiation, hydrogen peroxide or methyl methanesulfonate) or a treatment that causes a specific type of DNA lesion (e.g., the photosensitizer Ro19-8022 plus light, or potassium bromate treatment, to induce DNA oxidation). There are different assay controls for the standard alkaline comet assay and the enzyme-modified comet assay. The compound used for the enzyme-modified assay should not generate DNA strand breaks, as these decrease the dynamic range of the enzyme-sensitive sites. In biomonitoring studies, as well as cross-sectional, interventional and clinical studies, unexposed cells can be used as assay controls. It is ‘essential’ to report levels of damage in assay controls and assay variation (standard deviation) in both the standard and enzyme-modified comet assay.

The in vitro DNA repair assay uses internal experimental controls, which are also used in the calculation of the repair activity, rather than assay controls per se. It is ‘essential’ information to report the level of DNA repair incisions in nucleoids from (i) non-exposed substrate cells incubated with reaction buffer, to determine the basal level of DNA damage in the substrate DNA (i.e., the ‘background control’); (ii) exposed cells incubated with the reaction buffer, to reveal the level of any nonspecific DNA strand breaks or abasic sites resulting from the treatment with the damaging agent (i.e., the ‘treatment control’); (iii) non-exposed substrate cells incubated with protein extract from the sample, to check for nonspecific incision or cleavage activity (i.e., the ‘specificity control’); and (iv) exposed substrate cells incubated with lesion-specific enzyme, similar to assay controls for the enzyme-modified comet assay (i.e., the ‘incubation reaction control’).

#### Step 1D: Negative and positive controls

In this article, negative and positive controls refer to the experimental groups, as described in the OECD guideline (TG 489) for the in vivo comet assay in animal tissues^18^. Thus, negative and positive controls pertain to the whole experiment. A positive control refers to a direct- or indirect-acting genotoxic compound that produces DNA strand breaks or enzyme-sensitive sites detected with the comet assay. For the enzyme-modified comet assay, there is no list of positive controls available that corresponds to the compounds the OECD lists as positive controls for the alkaline comet assay (for inducing DNA strand breaks) in specific animal tissues. In addition, a positive control does not exist for human biomonitoring studies, as healthy people cannot deliberately be exposed to a genotoxic agent. Positive controls are already considered mandatory in cell culture experiments in genetic toxicology and will de facto be ‘essential’ information in articles using the comet assay. In animal studies, however, for practical purposes comet data on assay controls (i.e., samples mentioned in the section above entitled ‘Step 1C, Assay Controls’) are sufficient, whereas results from true negative and positive controls are considered to be ‘desirable’ information only.

#### Step 2: Embedding of the cells in agarose

The comet assay was originally developed using three layers of agarose on glass slides, with the middle layer containing the cells. However, the top layer of agarose is not necessary, and certain procedures do not use a bottom layer (e.g., Gelbond film assays). It is ‘desirable’ to report the type of slides and size (i.e., surface area) of the gels, as the proportion of the cells near the edge of the gel increases as the gel size decreases and the DNA in nucleoids at the edge of the gel may migrate differently from that in nucleoids toward the center of the gel^22^. The final concentration of agarose (with the cells embedded therein) is ‘essential’ to report, as the migration of DNA depends on the density of the gel.

#### Step 3: Lysis of the cells

There are several procedures for lysing cells in the comet assay. Information about the composition of the lysis solution is ‘essential’. An extra enzyme incubation step (e.g., with proteinase K) may be required for certain types of cells, and details of the incubation should also be reported as ‘essential’ information. Some reports suggest that the duration of lysis may affect the stability of certain types of DNA lesions^28–30^, so the duration of the lysis and temperature of the lysis solution are ‘desirable’ information to report.

#### Step 4A: Repair enzyme treatment in the enzyme-modified comet assay

As the lysis solution may inhibit the activity of the repair enzyme, it is ‘desirable’ to establish whether a washing step was performed between the lysis step and enzyme treatment (i.e., composition of the washing buffer, number of washings and duration). It is ‘essential’ to relay information about the source of repair enzymes, as enzymes from different manufacturers have been shown to differ in both their activity and their specificity towards nucleobase lesions^16^. In most comet studies, titration curve experiments are performed to identify optimal conditions for the enzyme treatment^31^; however, the results of enzyme titration curves are rarely reported and are classified here as ‘desirable’ information (reference to a previous study could be made instead), although we regard it as ‘essential’ to report the duration and temperature of the treatment. The concentration of enzyme applied to the gel is ‘essential’ information; it is preferable to report the concentration in enzymic units (U/ml), although the protein concentration (mg/ml) is also useful. The type of incubation unit (e.g., incubator, slide moat or 12-gel system) and mode of incubation are ‘desirable’ information to report. It should be stated whether the incubation was performed (i) in an enzyme bath or with a drop and coverslip on the slide, (ii) in a standard 2-gel version, 12-gel or multiple-well system or (iii) in a humidified box in an incubator, on a heating plate or in a heated slide moat.

#### Step 4B: Extract preparation and incubation for the in vitro DNA repair assay

It is ‘desirable’ to report the number of cells or mass of tissue used to prepare the protein extracts, whereas it is ‘essential’ to report the final protein concentration of cell or tissue extracts that is added to the substrate DNA. The composition of the extraction buffer (‘desirable’ information) is less crucial than the composition of the incubation buffer (‘essential’ information), because the latter can affect the background level of DNA migration. In keeping with the information for the enzyme-modified comet assay, it is ‘desirable’ to report the results from titration experiments or reference previous studies from the same laboratory, where these have been performed. The volume of extract added to the gel-embedded substrate cells is ‘desirable’ information. It is ‘essential’ to report the duration and temperature of the incubation period because these affect the number of repair incisions. As for the enzyme-modified comet assay, the mode of incubation is ‘desirable’ information to report for the in vitro repair assay. Information on the identity of the enzyme used as the incubation reaction control (indicating the amount of DNA lesions in the substrate cells) and the composition of the buffer used for the background level of DNA repair incisions in unexposed substrate cells are ‘essential’, because they are key to demonstrating the reliability of the DNA repair incision activity.

#### Step 5: Alkaline treatment

The high-alkaline-pH solution disrupts the hydrogen bonding that holds the DNA strands together and also converts certain nucleobase lesions into DNA strand breaks. Thus, the duration of alkaline treatment can affect the level of DNA migration in the subsequent electrophoresis^32–34^. Specific information concerning the composition of the alkaline solution, pH, temperature and duration of the treatment are thus ‘essential’ information to report.

#### Step 6: Electrophoresis

The most important drivers of DNA migration are the duration of electrophoresis and the electrical potential (voltage drop across the electrophoresis tank platform)^35^. It is ‘essential’ that the composition of the electrophoresis buffer, strength of electrophoresis (voltage gradient (V/cm) over the electrophoresis tank platform) and duration of electrophoresis are reported. High temperature during electrophoresis may affect the DNA migration^36^; thus, information about the temperature of the electrophoresis solution is ‘essential’, and this may be accompanied by descriptions of steps taken to keep the temperature constant (e.g., cooling the platform or circulating the buffer).

#### Step 7: Neutralization

This step involves removing excess alkaline solution from the slides to ensure efficient staining. It is ‘desirable’ to describe the composition of the neutralization solution.

#### Step 8: Staining and visualization

DNA-binding dyes have differing binding affinities to DNA and may therefore affect the calculation of the primary comet assay descriptors in image analysis software differently^36–38^. The type of dye is ‘essential’ information, whereas the concentration is ‘desirable’ information, as is the time between staining and visualization. The intensity of the light from different types of microscope lamps differs. In addition, it varies due to the age of the lamp and the time it has been turned on during the scoring of comets. However, there is no standard procedure for measuring the intensity of light and correcting the level of DNA migration accordingly. Furthermore, the same comet may appear to have different levels of DNA migration at different microscope magnifications. Thus, it is ‘desirable’ to report the microscope magnification used for the scoring. It is ‘desirable’ to show representative images of comets (e.g., control with little or no migration, moderate and extensive DNA migration) alongside the reported levels of DNA migration.

#### Step 9A: Scoring and data analysis

This step requires the reporting of ‘essential’ information for the primary comet assay descriptor (e.g.. %DNA in tail, tail length, tail moment or visual score), the number of comets that are analyzed per sample and how the overall level of DNA migration is expressed (e.g., median or mean of comet scores). It is ‘not important’ to report the individual result of each comet in each gel; they are combined to calculate the overall damage level. As it cannot be ruled out that different image analysis software packages may have different ways to calculate the primary comet assay predictor, it is ‘essential’ to report the software used (software name, manufacturer, version). All primary descriptors share the limitation that it is necessary to have expertise in the comet assay to understand what they mean, whereas if a calibration curve is created (using ionizing radiation, which induces breaks at a known frequency), results can be converted to relative lesion frequency compared to unaltered nucleotides or nucleobase pairs; such data are unequivocal and convey information that all researchers can understand^39^. The procedure to obtain a calibration curve, using ionizing radiation, and convert DNA migration levels to the lesion frequency relative to unaltered nucleotides or nucleobase pairs has been reported previously^40^. Thus, reporting the results as levels of lesions per 10^9^ unaltered nucleotides or 10^6^ nucleobase pairs is ‘desirable’.

#### Step 9B: Calculation of enzyme-sensitive sites or net number of incisions in substrate DNA for the in vitro DNA repair assay

The incubation with DNA repair enzymes increases the level of DNA migration, as both the basal level of DNA strand breaks and enzyme-specific lesions contribute to the total number of DNA strand breaks. As the DNA migration that is attributed to the basal level of DNA damage and enzyme-specific lesions may originate from different mechanisms of genotoxicity, it is insufficient to report only the total level of DNA damage after enzyme treatment. Instead, it is ‘essential’ to report the genotoxicity as enzyme-sensitive sites, where the basal level of DNA migration has been subtracted from the DNA migration in the enzyme-treated slides.

As the in vitro DNA repair assay uses the same substrate cells with all cell or tissue extract samples, it is unnecessary to have concurrent background and treatment controls for each sample in the same experiment (i.e., assay run). There might be more than one substrate (e.g., when analyzing both base- and nucleotide-excision repair activity), and therefore separate controls for each type of DNA repair assay are needed. It is ‘essential’ to report the net incisions by the repair extract in the substrate DNA.

#### Step 9C: Statistical analysis of the results

Statistical analyses are ‘essential’. The type of statistical analysis depends on the design of the study and follows the general assumptions in statistical testing in genetic toxicology and biomonitoring^41,42^.

## Conclusion

All articles that include results from comet assays should have a clear description of the experimental protocol. In principle, they should contain as much information about the comet assay procedure as possible, but some details are more important than others. Most journals with a word count limitation have supplementary sections where the details of the procedure can be described. However, if this is not possible, authors should cite articles with a detailed procedure (preferably those that are open access or freely available). Terms such as ‘modified from’ and ‘adapted from’ should be avoided, unless the specific modifications are defined.

The MIRCA recommendations represent a standardized reporting checklist for the description of comet assay procedures and results. However, this is not a guide to best-practice procedures for the assay. The MIRCA recommendations provide an important tool to aid researchers, reviewers and editors in ensuring that the comet assay is performed rigorously and reported comprehensively. Taken together, these will increase the quality and impact of comet assay results in scientific studies.

## Supplementary information

Supplementary Table 1 Supplementary Table 1.
